# Successful adaptation of a research methods course in South America

**DOI:** 10.1080/10872981.2017.1336418

**Published:** 2017-06-19

**Authors:** Leonardo Tamariz, Diego Vasquez, Cecilia Loor, Ana Palacio

**Affiliations:** ^a^ Department of Medicine, Miller School of Medicine, University of Miami, Miami, FL, USA; ^b^ Veterans Affairs Medical Center, Miami, FL, USA; ^c^ Facultad de Ciencias Medicas, Universidad Catolica Santiago de Guayaquil, Guayaquil, Ecuador; ^d^ Vicerectorado Academico, Universidad Catolica Santiago de Guayaquil, Guayaquil, Ecuador

**Keywords:** Research methods, practice based, flipped classroom, curriculum, South America

## Abstract

**Background**: South America has low research productivity. The lack of a structured research curriculum is one of the barriers to conducting research.

**Objective**: To report our experience adapting an active learning-based research methods curriculum to improve research productivity at a university in Ecuador.

**Design**: We used a mixed-method approach to test the adaptation of the research curriculum at Universidad Catolica Santiago de Guayaquil. The curriculum uses a flipped classroom and active learning approach to teach research methods. When adapted, it was longitudinal and had 16-hour programme of in-person teaching and a six-month follow-up online component. Learners were organized in theme groups according to interest, and each group had a faculty leader. Our primary outcome was research productivity, which was measured by the succesful presentation of the research project at a national meeting, or publication in a peer-review journal. Our secondary outcomes were knowledge and perceived competence before and after course completion. We conducted qualitative interviews of faculty members and students to evaluate themes related to participation in research.

**Results**: Fifty university students and 10 faculty members attended the course. We had a total of 15 groups. Both knowledge and perceived competence increased by 17 and 18 percentage points, respectively. The presentation or publication rate for the entire group was 50%. The qualitative analysis showed that a lack of research culture and curriculum were common barriers to research.

**Conclusions**: A US-based curriculum can be successfully adapted in low-middle income countries. A research curriculum aids in achieving pre-determined milestones.

**Abbreviations**: UCSG: Universidad Catolica Santiago de Guayaquil; UM: University of Miami

## Introduction

In the USA, pre-graduate and graduate medical participation in research is common, and this involvement is mutually beneficial to universities, mentors, and students [1].

A variety of research curriculums for residents have been implemented over the past few years, with programs reporting that a structured research curriculum can substantially enhance scholarly success for physicians-in-training and their mentors [2–5].

In South America, in comparison to other countries, research productivity is low. Factors that have been related to this lack of scholarly work are: a lack of a structured curriculum [6,7], lack of English proficiency [7], and a lack of knowledge of research methods [8].

The aim of this study is to report our experience adapting an active learning-based research methods curriculum to improve research productivity at a university in Ecuador.

## Methods

### Setting and population

We used a mixed-method approach to evaluate an adapted research curriculum at the Universidad Catolica Santiago de Guayaquil (UCSG), in Guayaquil, Ecuador. The UCSG is a large not-for-profit institution with approximately 17 500 students. We conducted two research methods courses during two consecutive years (2015 and 2016). A month before each of the courses took place, we asked a medical school champion to promote the course and facilitate the creation of teams. Each team included six to seven members, amongst them: medical students in their final year, postgraduate trainees, and at least one university professor per team. Each team had a specific specialty theme area. The rationale for the creation of groups was two-fold: (1) Expose trainees to teamwork that could facilitate the conduction of research in low resource environments, and (2) Given the intensive brief format of the course, have a manageable number of topics that would allow hands-on mentoring. The University of Miami institutional review board approved this study.

### Description of the research methods course

This program was an adaptation of our program at the University of Miami (UM). Briefly, our UM program uses a flipped classroom, active learning approach, with online educational modules before each large group session, and one-on-one mentoring sessions. Its duration is between six months to three years, depending on the residency program. The outcome of our program was the presentation in a national meeting or publication in a peer-reviewed journal [5,9].

The key consideration in the adaptation process was delivering the course in an intensive format. Given the practical hands-on approach of our educational model, we decided that an intensive course format targeting research teams or groups, rather than individuals, was beneficial. Additionally, in contrast to our UM model, we requested that the mentor attend the workshop with his or her team. The purpose was three-fold: (1) To serve as a role model in research and mentoring for the local faculty, (2) Create a team identity with a faculty champion, and (3) Have their feedback regarding feasibility, sources of data, and relevancy of the project, readily available throughout the course.

Table 1 describes the course. The research methods course at UCSG was a four-day, 16-hour course, followed by monthly skype mentoring meetings with each team throughout a six-month period, to monitor progress and provide feedback. The four-day course covered the development of a research question, study design, literature search, and the definition of variables, bias, confounding variables, and statistical analysis. The course used a combination of novel approaches to improve research competence. The adaptation was based on an active experiential learning approach that used elements of problem-based learning and practice-based learning for certain sections. We worked on each key concept of the course using the following three-step strategy:

1. The instructors presented content and practical examples of each key concept (e.g. characteristics of a good research question). This replaced the online videos used in our UM curriculum, in accordance to the flipped-class model.
2. Each team worked on this concept for its own research idea (fine tuning their own research question). Each team had computers and internet access accessibility in order to search for information as needed. For certain sections, we confronted teams with a logistical or methodological problem and asked them to come up with possible solutions (e.g., sampling strategy to reduce selection bias, low expected event rate, risk of loss to follow-up of the selected target group, low literacy among survey recipients, etc.)
3. Each team presented their work to the entire group and provided feedback to each other. This group discussion facilitated the discussion of methodological concepts and questions, and facilitated improvement in the concrete outputs of each team.

**Table 1.** Description of the course.

Day	Objectives	Educational strategy
1	Formulate a research question Discuss study design	Brief powerpoint of key concepts 1:1 meetings to evaluate the quality of the research question Practice based learning of abstracts to determine the study design. Group discussion and feedback
2	Conduct literature search Definition of variables	Practice-based learning searching for Pubmed 1:1 meeting to review the literature search Brief lecture of key concepts of bias on blackboard Practice-based learning to identify the different types of bias
3	Statistical analysis	Brief lecture of key concepts on blackboard Practice-based learning to understand basic and complex statistical analysis with a mock study 1:1 meeting to discuss analysis plan
4	Writing	Brief lecture of key concepts on blackboard How to write the different sections of a manuscript Practice based learning on how to write a manuscript

### Outcomes

#### Scholarly success

We defined scholarly success as the presentation of group research results in a South American or US national meeting, or the publication of the work in peer-review journals. Abstract submission was verified by collecting acceptance letters, and publications in peer-reviewed journals were verified by conducting a MEDLINE search in February 2017 or by acceptance letters from journals.

#### Change in research methods knowledge and perception of competence

We collected a survey amongst all participants up to 30 days before and after the course to compare knowledge and perception of research competence. We measured knowledge as the percentage of correct answers to a previously validated survey [10].

We asked, ‘How prepared do you feel in your ability to conduct research?’ as a perception of competence on a five-point Likert scale ranging from ‘Somewhat’ to ‘Very prepared.’

#### Satisfaction

We asked the course participants, ‘How satisfied are you with the course?’ with responses measured on a five-point Likert scale ranging from ‘not satisfied’ to ‘completely satisfied.’ We defined satisfaction as participants who responded, ‘completely satisfied’.

### Qualitative methods

We conducted key informant interviews after course completion using video recordings of selected faculty and student members of each group. Two investigators (LT and DV) reviewed the videos and organized the quotes as themes, stratifiying the comments by faculty or students. A third investigator decided on disagreements between the two investigators (CL). The interviews attempted to discuss barriers to conducting research and their perception of the research methods course.

### Statistical analysis

We reported categorical variables as percentages and continuous variables as medians with interquartile range. To compare the pre- and post- change in competence and knowledge, we reported p-values using the paired t-test. All analyses were two-tailed and were conducted using STATA 14 (College Station, USA).

## Results

### Baseline characteristics

Fifty university students and 10 faculty members attended the course. Table 2 reports the baseline characteristics of the participating groups. All students who signed up for the course attended, but four faculty members who had agreed to participate did not. None of the students who participated in course 1 participated in course 2 and vice versa, and only one faculty member participated in both. We had fifteen groups participating in the course. Seventy-three percent were from medical specialties while the remaining groups were from public health and social work. Seventy percent of the groups had virtual follow-up mentoring meetings.

**Table 2.** Baseline characteristics of participating groups.

Area of research	Number of students	Number of faculty
Course 1		
Neurology	7	1
Obstetrics and gynecology	5	1
Public health	5	1
Ophthalmology	2	1
ENT	2	0
Cardiology	5	1
Course 2		
Neurology	5	1
Public health 1	3	1
Nephrology	2	0
Public health 2	4	0
Obstetrics and gynecology	4	0
Social work	7	2
Pediatrics	3	1
Rheumatology	4	1
Gynecology	5	1

### Research methods knowledge

**Figure 1.**
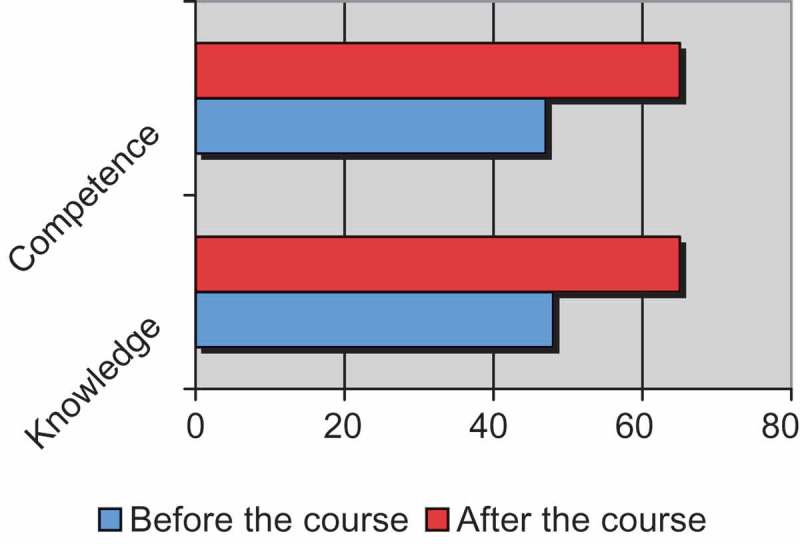
Measures before and after the course.

Figure 1 shows knowledge assessments before and after course completion. The mean percentage of correct answers before the test was 48% compared to 65% correct answers after course completion (p = 0.02).

### Perception of competence

Figure 1 shows the perception of confidence before and after course completion. The mean percentage of ‘somewhat’ or ‘very prepared’ was 47% before the course compared to 65% after the course completion (p = 0.01).

### Satisfaction

The percentage of participants who thought the objectives of the course were met was 74%, and the percentage of participants who would recommend the course to other students was 100%.

### Scholarly work

Table 3 summarizes the research productivity by group. The presentation or publication rate for the entire group was 47%. All groups that presented or published its work had at least four virtual follow-up mentoring meetings, whereas those who did not publish only had zero to two follow-up meetings.

**Table 3.** Research productivity by groups.

Area of research	Presentations	Publications	Grants
Course 1			
Neurology	5	3	1
Obstetrics and gynecology	0	0	0
Public health 1	2	1	1
Ophthalmology	1	0	0
ENT	0	0	0
Cardiology	0	0	0
Course 2			
Neurology	0	0	0
Public health	1	1	0
Nephrology	1	1	0
Public health 2	1	1	0
Obstetrics and gynecology	0	0	0
Social work	0	1	1
Pediatrics	0	0	0
Rheumatology	0	0	0
Gynecology	0	0	0

### Qualitative results

Table 4 reports the qualitative results. We identified three clear themes. Barriers to conducting research was the most commonly mentioned theme. Faculty members cited the lack of research culture, the lack of expertise in clinical research, and institutional barriers for starting a research project. Students viewed the lack of a structured but practical curriculum as a key factor in the fear and misconceptions regarding challenges conducting research. The common themes mentioned regarding the course were that both faculty members and students enjoyed the format of the course, and also that course goals were appropriate. Students identified having a strong faculty leader as a key to success.

**Table 4.** Qualitative results.

Themes	Faculty	Students
Barriers	No research culture in Ecuador	Afraid of a challenging field
	Lack of faculty with expertise on research	Misconception of research
	Difficulty with data collection	Medical students have an aversion to numbers
	Institutional barriers to obtaining and executing grants	Lack of a curriculum
Faculty involvement in the course	Enjoyed working with the students	Faculty leaders were the key to success
Format of the course	Enjoyed interaction and group work	Enjoyed the practical sessions
		Enjoyed minimizing PowerPoint

## Discussion

Our study found that an in-person intense 16-hour curriculum using an active experiential learning approach not only increases knowledge and perception of competence but also increases scholarly activities. The study also identified important barriers to conducting research in Ecuador, and keys to success when starting a project. The strengths of this study are the use of a strict definition of scholarly success and the use of qualitative methods to evaluate achievement of educational goals.

The main limitation of our study is the lack of a formal comparison group to assess curriculum effectiveness. However, a search for faculty/student publications related to UCSG was conducted during the same time period and only yielded publications related to the course. The second limitation is the small sample size since we have only been able to accommodate two courses and report the outcomes for both. The third limitation is that we could not use a more formal evaluation metric such as the consolidated implementation framework or case-studies, since the curriculum was not fully adopted by the university.

Our flipped classroom curriculum in the USA for postgraduate training has been successful in increasing academic productivity and research quality [5].

That model encompasses elements that make a curriculum successful, such as having research directors, protected time, research days, and faculty involvement [11].

The adaptation of the curriculum was challenging because the intense four-day format limited the amount of work possible between sessions and the use of our flipped classroom model. In our US-based format, learners have days or weeks in between sessions to view online materials and work through the different sections or stages of the project. We successfully addressed this issue by: (1) briefly providing content at the beginning of each section, (2) by creating research teams that could share the workload and produce a deliverable within the time constraints, and (3) by having virtual skype follow-up mentoring meetings to provide feedback to each team as the work progressed. A key challenge for several groups was local faculty member disengagement after the four-day workshop.

Our adapted curriculum uses a combination of novel elements from a variety of models. We used an active learning approach throughout the course to maximize learning by a hands-on approach, as it is more effective for competence acquisition. However, for certain sections, we also used problem or practice-based approaches. This was done to increase their analytical engagement, while also providing them with confidence in their ability to find potential solutions and more importantly, in their ability to ask the correct questions. Students and faculty enjoyed the format and were able to identify key threats to validity and potential solutions for each of the studies. This experience suggests that intensive curriculums with limited in-person instructor contact are a feasible strategy to improve research competency in Latin American universities. This makes the dissemination of research curriculums for medical students, post-graduate trainees, and faculty, a distinct possibility.

The results regarding perception of competence deserve further comment. Self-perception of competence is the reported ability of performing a task [12]. In our study, the majority of the learners felt that the course helped them do scholarly work. The objective of our course was to achieve perceived competency as an initial stepping stone towards scholarly success. This is important since it has previously been noted that perceived competence is correlated with real competence [13].

An important result of this study was that groups who achieved scholarly success had a strong faculty member who attended all the sessions and continued to work on the study after the course was delivered. This result is similar to our prior results in the U.S, where the publication or presentation at a national meeting was only predicted by the productivity of the mentor. This is not surprising, given the fact that the process of publication requires perseverance and skills that are more likely to be present amongst mentors who are motivated to conduct research. Universities considering implementing a research curriculum should identify local faculty champions and incentivize their participation in the research projects. Group work was beneficial for several reasons. First, developing mentor-mentee relationships in the context of structured research programs, is beneficial for both residents and faculty members. Second, even in programs with little clinical research infrastructure, providing a basic structure and few resources can harness the potential already present among the faculty. Third, brief faculty development initiatives to improve their skills in selecting a publishable topic, developing a manageable research question, preparing an abstract or manuscript, and responding to requests of reviews, could have a significant impact in the number of projects that reach publication stages and could create a cycle of improvement for faculty and residents alike. Additionally, it has been shown that trainees who report having had an influential mentor are significantly more likely to mentor others [14].

As previously reported by others regarding learning and a variety of fields, we have identified mentoring as a critical portion of our curriculum. Previous reports have shown that mentoring is invaluable for career development in academic medicine and is essential for mentees to develop confidence in his or her work. It facilitates career selection, career advancement, publication productivity, and achievement of grant funding [15,16].

Ideally, it is a dynamic, collaborative, reciprocal relationship focused on a mentee’s personal and professional development. This active learning-based curriculum facilitated the development of such relationships amongst faculty members and students who attended.

Universities interested in starting organized research need to identify research champions that can help teams of investigators navigate the difficult process, ranging from the start of a research project all the way to publishing the work. At the same time, universities need to incorporate a research curriculum into their syllabus to create a longitudinal curriculum. The role of mentorship is critical to the success of this process and alongside to identifying champions, universities need to create a pool of mentors that can guide students.

In conclusion, an active learning-based research methods curriculum, delivered in an intensive format with virtual follow-up, can be implemented in Latin America to increase research interest, competence, and productivity amongst health professionals at different stages of their careers. Future work should focus on developing feasible dissemination strategies and inter-institutional collaboration models across countries and regions.
